# The discovery, structure, and function of 5-HTR1E serotonin receptor

**DOI:** 10.1186/s12964-023-01195-0

**Published:** 2023-09-18

**Authors:** Vinay Kumar Sharma, Y. Peng Loh

**Affiliations:** Section On Cellular Neurobiology, Eunice Kennedy Shriver National Institute of Child Health and Human Development, National Institutes of Health, NICHD, NIH, 49, Convent Drive, Bldg 49, Rm 6A-10, Bethesda, MD 20892 USA

**Keywords:** 5-HTR1E, Serotonin, GPCR, G proteins, Cell signaling

## Abstract

Serotonin (5-hydroxytryptamine, 5-HT) is a unique neurotransmitter which can regulate various biological processes by activating thirteen different receptors. These serotonin receptors are divided into seven different classes based on their structure and functions. Since these receptors co-express in various tissue and cell types and share the same ligand (5-HT), it has been a challenge for the researchers to define specific pathway and separate physiological role for each of these serotonin receptors. Though the evidence of operational diversity of these receptors is continuously emerging, much work remains to be done. 5-HTR1E is a member of 5-HT1 receptor family which belongs to G-protein coupled receptors (GPCRs). Even after three decades since its discovery, 5-HTR1E remains the least explored serotonin receptor. Very high similarity with another family member (5-HTR1F) and its non-existence in mice or rats makes 5-HTR1E a difficult target to study. Despite these challenges, recent findings on the role of 5-HTR1E in neuroprotection and diseases such as cancer, have excited many researchers to explore this receptor in detail. Here, we provide the first review of 5-HTR1E, since its discovery in 1989 to 2023. We highlight the structural and functional characteristics of this important serotonin receptor in detail and propose future directions in developing 5-HTR1E as a drug target.

Video Abstract

**Supplementary Information:**

The online version contains supplementary material available at 10.1186/s12964-023-01195-0.

## Introduction

Serotonin, a monoamine neurotransmitter first discovered by Maurice Rapport [1, 2] has thirteen serotonin receptors known till date. Since the discovery of first serotonin receptor in guinea pig [3], these serotonin receptors have been identified in various animal species (see review [4]).The effects of serotonin (5-HT) are mediated by these receptors which has been further categorized into 6 distinct classes of G protein-coupled receptor (GPCRs) 5-HT1, 5-HT2, 5-HT4, 5-HT5, 5-HT6 and 5-HT7 and a ligand gated ion channel, 5-HT3 [5, 6].

The 5-HT1 receptor family is the largest class of serotonin receptor subtypes and belongs to GPCRs which are also known as 7 transmembrane (7TM) domain receptors. This major group is subdivided into 5 receptor subtypes named 5-HT1A, 5-HT1B, 5-HT1D, 5-HT1E and 5-HT1F. 5-HT1 receptors were initially grouped because of their high affinity for 5-HT [7] but they also exhibited a high affinity for an agonist, 5-carboxamidotryptamine (5-CT), except for 5-HT1E and 5-HT1F [8, 9]. All 5-HT1 receptor family members share high sequence homology (between 40–60%) with each other (see review [9] and Table 1) and also couples with Gαi/Gαo proteins to inhibit adenylyl cyclase (AC) activity (see review [4]). These overlapping similarities among 5-HT1 members provide the good reasons for their affiliation to the same receptor class while they are involved in different biological processes such as cell survival, neuronal regulation and neuroprotection [10–12] (ref. 10, review), CNS disorders, pain, depression [13–16], migraine [17] (re. 17, review) and cancer [18, 19] (ref. 18, review).

**Table 1:** Overall and transmembrane domains (TM) Sequence similarities (%) of 5-HTR1E with serotonin (5-HT) receptor subtypes. Receptor subtypes in bold are available for experimental structures. Reproduced with permission from Sharma et al., 2021 [12]

#	Subtypes	All	TMs	TM1	TM2	TM3	TM4	TM5	TM6	TM7
1	5-HTR1E	100.00	100.00	100.00	100.00	100.00	100.00	100.00	100.00	100.00
2	5-HT1A	38.90	52.91	23.81	62.50	52.38	38.89	78.26	61.90	52.63
3	**5-HT1B**	46.58	65.28	47.62	70.83	76.19	61.11	60.87	66.67	73.68
4	5-HT1D	45.48	66.69	38.10	70.83	76.19	72.22	73.91	61.90	73.68
5	5-HT1F	56.44	73.77	38.10	87.50	76.19	77.78	86.96	76.19	73.68
6	5-HT2A	27.67	41.85	38.10	45.83	47.62	55.56	30.43	33.33	42.11
7	**5-HT2B**	25.21	39.63	23.81	50.00	47.62	44.44	26.09	38.10	47.37
8	**5-HT2C**	27.67	44.26	38.10	54.17	52.38	44.44	34.78	33.33	52.63
9	5-HT4	29.32	45.32	28.57	58.33	47.62	38.89	47.83	38.10	57.89
10	5-HT5A	29.59	45.36	23.81	58.33	38.10	44.44	47.83	52.38	52.63
11	5-HT6	25.75	38.49	28.57	33.33	47.62	27.78	26.09	42.86	63.16
12	5-HT7	33.15	52.48	33.33	54.17	52.38	61.11	60.87	47.62	57.89

5-hydroxytryptamine receptor 1E (5-HTR1E) is the fourth member of the 5-HT1 receptor family which was identified about three decades ago. It is the only 5-HT1 member which does not express in mice or rat [20] and due to high similarity with another family member (5-HTR1F, which was not discovered then) was not recognized correctly. 5-HTR1F which was identified much later has been studied well and its role in migraine is well studied [17, 21, 22] while 5-HTR1E after its initial discovery without specific functions, remained largely unknown. Reports published prior to 2021 were mainly about the signaling and distribution of 5-HTR1E, but no physiological role was found for this receptor. Recently, a few studies have been published that indicate a role of 5-HTR1E in cell survival, neuroprotection, and cancer progression [11, 12, 19]. In addition to serotonin-induced classical pathways, 5-HTR1E can also function with a binding partner CPE/NFα1 protein to activate a non-canonical signaling pathway which makes this receptor more interesting [12]. Taken together, these new findings reveal the emerging roles of 5-HTR1E and are summarized in this review. Here, we cover the journey of 5-HTR1E since its identification and highlight its biological and pharmacological properties.

### Identification of 5-HTR1E

The introduction of radioligand binding studies in 1970’s became a key tool in identification of novel receptors. Since then, radiolabeled 5-HT or 5-CT has been widely used in pharmacological characterization of serotonin receptors. In the absence of many DNA or protein sequencing techniques, nomenclature of serotonin receptors also relied heavily on radioligand based identification of novel sites. Similarly, 5-HTR1E was also discovered using radio-labelled serotonin, [3H]-5-HT in human brain tissues [8]. In this study, the blocking agents for other 5-HT1 subtypes 5-HT1A, 5-HT1B,5-HT1C (now 5-HT2C), which had been identified in 1988, showed a biphasic displacement curve to 5-CT [8]. High affinity sites for 5-CT were thought to represent the 5-HT1D receptor while low affinity sites with novel pharmacology were assumed to be a new 5-HT_1E_ (5-HTR1E) receptor [8]. Later this radiolabeled 5-HT binding site (which was 5-CT-insensitive) was also found in guinea pig, rabbit, and dogs [8, 23, 24]. Since 5-HTR1F receptor which also have high affinity for 5-HT but 5-CT insensitive [22] was not discovered at that time, also contributed to the primary quantification of 5-HTR1E binding sites determined in above studies. Detailed analysis of overlapping 5-HTR1E binding sites in subsequent studies determined that the initial discovery of 5-HTR1E was a mixture of other 5-HT receptors like 5-HTR1E and 5-HT6 [20, 25].

### Cloning of *5-HTR1E* gene

Following the identification of 5-HTR1E in human brain tissues, a gene encoding a protein with 5-HT1 receptors pharmacology was cloned by Levy et al., from a human lambda EMBL3 genomic library and referred as *S31* [26]. By expressing in murine Ltk-cells they reported that S31 gene encodes a functional protein (did not refer as 5-HTR1E) which can mediate a 5-HT induced decrease in forskolin-stimulated cAMP and has the same pharmacological profile as the other 5-HT1 receptors. [26]. Within a month McAllister et al., published another report where they used degenerated primers (referred as AC1) based on the conserved structure of GPCRs to isolate cDNA clones encoding putative GPCRs from a human hippocampal cDNA library. In this study degenerated primers devised from transmembrane regions III and VI of the 5HT1A receptor, substance K receptor, the α2-adrenergic, βl-adrenergic, and β-adrenergic receptors were used to clone *5-HTR1E* gene [27]. Soon after, another group isolated the same *S31* gene from a human placental genomic library by using the oligonucleotide probes derived from transmembrane regions of the cloned human 5-HT1D_β_ (old name) receptor and ultimately confirmed that human *S31* gene encodes the pharmacologically defined serotonin 5-HTR1E receptor [28]. Both groups [26, 28] reported that 5-HTR1E shares high similarity with the 5-HT1Dα and 5-HT1Dβ (now 5-HT1B and 5-HT1D) receptors and it can inhibit adenyl cyclase function ultimately leading to reduction in cAMP levels. In the following years several reports claimed to have identified 5-HTR1E in mouse and rat [29, 30] but they were actually characterizing a putative receptor which was later identified as 5-HTR1F [22] as it wasn’t clear until Bai et al., confirmed the non-existence of 5-HTR1E gene in mouse or rat genome. This group first time reported the cloning of *5-HTR1E* gene from a small laboratory animal, guinea pig, while attempts to clone this gene from mouse or rat failed [20].

### Structural features of 5-HTR1E

The human S31 gene is located on human chromosome 6q14-q15 and does not have any intron, while the 5-HTR1E protein is 365 amino acids long [26, 27, 31] and shows a band of ~ 45 kDa on SDS-PAGE [12, 32]. Based on the sequence identity with other receptors and its hydrophobic profile 5-HTR1E was predicted to have 7 transmembrane helices which is a trademark of GPCRs [26, 28]. In comparison with other 5-HT1 family members, human 5-HTR1E receptor has highest homology with the 5-HTR1F (57.7%) followed by 5-HTR1B (46.58%), and 5-HTR1D (45.58%) receptors at amino acid level (Table 1).

The guinea pig 5-HTR1E receptor is also 365 amino acid long which shares 88% (nucleic acid) and 95% (amino acid) homology with the human receptor [20]. The other species where 5-HTR1E has been cloned is chicken and the 371 amino acids long protein also shares a high degree of sequence identity (83%) with humans [33]. The 5-HTR1E gene does not show any polymorphisms amongst humans [34].

### 5-HTR1E distribution

To date, only a few studies have been conducted on the expression and distribution of 5-HTR1E.

The expression of 5-HTR1E was first detected in human frontal cortex using autoradiographic studies [35] and then in the hippocampus of human post mortem brain homogenates [36]. It should be noted that these results might have been confounded by the labelling of 5-HT1F receptors. Bruinvels et al., also reported 5-HTR1E receptor mRNA in human and monkey brain sections using in situ hybridization technique, and high expression of 5-HTR1E was observed in human frontal cortex, putamen and globus pallidus [37]. qRT-PCR mRNA expression analysis in guinea pig showed that 5-HTR1E was present in brain with the highest expression in the hippocampus, followed by the olfactory bulb, while lower levels were detected in the cortex, thalamus, pons, hypothalamus, midbrain, striatum, and cerebellum. No expression of 5-HTR1E mRNA was found in liver, spleen, kidney, heart, lung, muscle, aorta, vena cava, and small intestine of the guinea pig [20]. Radioligand binding studies using [3H]5-HT in guinea pig hippocampal tissues also confirmed the high expression of 5-HTR1E in hippocampus and cortex tissues [32]. Later, this same group performed a detailed immunohistochemical analysis using 5-HTR1E specific antibodies and reported the specific localization of 5-HTR1E protein in guinea pig brain. The expression of 5-HTR1E was highest in olfactory glomeruli which was suggestive that 5-HTR1E might have a role in regulation of smell. In addition to the olfactory bulb, hippocampus and cerebral arteries were the only tissues with significant 5-HTR1E receptor expression. For 5-HTR1E distribution summary in brain, see Table 2. In hippocampus dentate gyrus (DG) has the most robust expression of 5-HTR1E while CA1, CA2 and CA3 showed low level of staining [25]. Recently, Sharma et al., reported the 5-HTR1E expression analysis in human hippocampal neurons where 5-HTR1E co-localizes with its interacting partner NF-α1/CPE. Immunohistochemical and immunofluorescence studies done on postmortem human brain sections showed that 5-HTR1E is localized in the perikarya of pyramidal neurons of CA1, CA2 and CA3 and within the granule cell layer of DG [12]. Since NF-α1/CPE is involved in memory and learning [38], a role of 5-HTR1E can be assumed in the regulation of these hippocampal functions via interaction with NF-α1/CPE. Brain cortex is involved in a wide range of neurological functions, including sensory and motor activities, vision, hearing, taste etc. [39] (book chapter) and almost all the studies listed in Table 2 report significant expression of 5-HTR1E in cortex tissues which suggests that HTR1E could be involved in some of these cortical functions. Since no experimental data is available regarding the role of 5-HTR1E in brain functions, such proposals will require further investigation.

**Table 2:** 5-HTR1E mRNA and protein distribution in various brain regions

**Anatomical region**	**Human** **5-HTR1E** **Ligand binding** (Lowther et al., 1992) [36]	**Human** **5-HTR1E** **Ligand binding** (Miller et al., 1992) [35]	**Human** **5-HTR1E** **in situ hybridization** (Bruinvels et al., 1994) [37]	**Guinea pig** **5-HTR1E** **PCR mRNA analysis** (Bai et al., 2004) [20]	**Guinea pig** **5-HTR1E** **Ligand binding** (Klein et al., 2009) [32]	**Monkey** **5-HTR1E** **in situ hybridization** (Bruinvels et al., 1994) [37]	**Chicken 5-HTR1E** **PCR mRNA analysis** (Sun et al., 2021) [33]
Hippocampus	** + + **			** + + + + **	** + + + + **		
Olfactory bulb				** + + + + **	** + + + + **		
Cortex	** + + + (F)**	** + + + (F)**	** + + + (V)**	** + + **	** + + + (F)**	** + (F)**	
Amygdala	** + + + **		** + + + **	** + + **			
Hypothalamus			** + + + **		** + + **		** + + **
Cerebellum				** + **	** + + **		** + **
Striatum				** + **	** + **		
Thalamus				** + + **	** + **		
Brain stem					** + **		
Midbrain				** + **			** + **
Pons				** + + **			
Globus pallidus	** + + **	** + + + **					
Caudate	** + + + + **		** + + + + **			** + + + **	
Putamen	** + + + + **	** + + + + **	** + + + **			** + + + **	
Pituitary							** + + + + **

Very High +  +  +  + , High +  +  + , Moderate +  + ,Low +

(F) Frontal cortex (V) visual cortex. Ligand binding: serotonin ligand

### 5-HTR1E pharmacology

In the first identification report on 5-HTR1E, [3H] labelled 5-HT was used for radioligand binding studies. Radiolabeled 5-HT displayed high affinity (Kd = 5.3 nM) and saturability (B_max_ = 83 fmol/mg) for 5-HTR1E receptor in human brain cortical tissues [8] (Table 3). During the competition studies with nonradioactive drugs 5-CT and ergotamine did not display the affinity for 5-HTR1E binding site but the interaction between 5-HTR1E and nonhydrolyzable derivatives of GTP, guanosine 5’-0-(3-thiotriphosphate) (GTPγS) and 5’-guanylyl-imidodiphosphate [Gpp(NH)p] inhibited the binding between [3H] labelled 5-HT and 5-HTR1E (with the IC50 values of 16 and 172 nM, respectively) while adenosine 5’-0-(3-thiotriphosphate) (ATPγS) and 5’-adenylyl-imidodiphosphate [App(NH)p] had no effect on this binding. Eventually, this high affinity for 5-HT and the interaction with a GTP-binding protein became the reason to designate 5-HTR1E as the fifth serotonin receptor keeping with the existing system of nomenclature for 5-HT receptors [8]. While human brain cortical tissues used for binding experiments were inclusive of 5-HTR1F (unidentified till then), another pharmacological evaluation of human 5-HTR1E was also done by using [3H] labelled 5-HT but with the stable expression of 5-HTR1E in murine fibroblasts and determined the binding affinity between 5-HTR1E and serotonin (Kd = 9.7 nM). Saturation binding experiments in these 5-HTR1E expressing membranes showed the B_max_ value (2.4 pmol/mg of protein) for [3H] labelled 5-HT while most of the other compounds tested had low affinity for 5-HTR1E protein (Kd > 200 nM). The affinity of other ligands for 5-HTR1E (descending order) was 5-HT > methysergide > ergotamine > 8-hydroxy-2-(di-n-propylamino) tetralin > 5-carboxyamidotryptamne > ketanserin, while 5’-Guanylyl-imidodiphosphate was able to decrease [3H] 5-HT binding in a dose-dependent manner [28] (Table 3). AC1 cDNA-encoded human 5-HTR1E receptor, transiently expressed in human embryonic kidney cell line (HEK293) also displayed high affinity (Kd = 15 nM) and saturability (15 to 40 pmol/mg of protein) when incubated with [3H] labelled serotonin. 5-CT showed low affinity (pKi = 5.15) compared with serotonin (pKi = 8.14) in competition with [3H] 5-HT which was consistent with the previous binding results of 5-HTR1E [27] (Table 3).

**Table 3:** Binding affinity of different compounds and drugs with 5-HTR1E receptor (Summarized from various studies)

**(Leonhardt et al., 1989)** [8]		**(McAllister et al., 1992)** [27]				
**Compound**	**Human cortex (pKi)**	**Compound**	**Transient expression (pKi)**	**Stable expression (pKi)**	**Human Frontal cortex, (pKi)**	
5-HT	3.5 ± 0.3	5-HT	8.14 ± 0.02	8.21 ± 0.08	8.23 ± 0.19	
Ergotamine	155 ± 32	5-CT	5.15 ± 0.03	5.48 ± 0.05	5.67 ± 0.09	
5-CT	910 ± 149	Methysergide	6.49 ± 0.04	6.66 ± 0.02	6.76 ± 0.09	
DOB	556 ± 60	Sumatriptan	5.63 ± 0.02	5.68 ± 0.08	5.89 ± 0.05	
TFMPP	570 ± 33	Metergoline	5.95 ± 0.03	6.11 ± 0.05	6.37 ± 0.11	
8-OH-DPAT	826 ± 33	Methiothepin	6.68 ± 0.02	6.92 ± 0.16	5.81 + 0.09	
Methysergide	59 ± 8	Ergotamine	6.24 ± 0.02	6.27 ± 0.09	6.10 ± 0.19	
**(Zgombick et al., 1992)** [28]			**(Bai et al., 2004)** [20]			
**Compound**	**Human 5-HTR1E (pKi)**	**Hill coefficient**	**Compound**	**Guinea pig (pKi)**		**Human (pKi)**
5-HT	11 ± 1	0.92 ± 0.01	5-HT	8.252 ± 0.036		8.216 ± 0.041
Lysergol	43 ± 5	0.92 ± 0.13	a-Methyl-5-HT	7.129 ± 0.025		7.017 ± 0.096
Ergonovine	88 ± 8	0.87 ± 0.05	Tryptamine	6.573 ± 0.035		6.532 ± 0.045
Methylergonovine	89 ± 4	0.92 ± 0.04	5-Methoxytryptamine	6.379 ± 0.047		6.237 ± 0.026
αMe-5-HT	121 ± 13	0.84 ± 0.04	5-Fluorotryptamine	6.387 ± 0.016		6.301 ± 0.044
Methiothepin	194 ± 4	0.87 ± 0.03	Sumatriptan	5.742 ± 0.021		5.809 ± 0.030
1-Naphthylpiperazine	207 ± 69	1.11 ± 0.08	5-CT	5.500 ± 0.029		5.268 ± 0.029
Methysergide	228 ± 16	0.91 ± 0.07	1-Naphthylpiperazine	7.158 ± 0.040		7.010 ± 0.045
Oxymetazoline	419 ± 49	0.80 ± 0.08	DOI	5.535 ± 0.032		5.754 ± 0.043
5-MeO-OMT	528 ± 32	1.05 ± 0.10	8-OH-DPAT	5.516 ± 0.022		5.501 ± 0.012
Ergotamine	599 ± 39	1.02 ± 0.04	*m*-CPP 69	5.513 ± 0.088		5.424 ± 0.089
2-Me-5-HT	817 ± 203	0.86 ± 0.06	TFMPP	5.277 ± 0.079		5.342 ± 0.060
Yohimbine	1270 ± 233	0.93 ± 0.11	Ergonovine	7.249 ± 0.209		7.287 ± 0.155
Sumatriptan	2520 ± 135	0.92 ± 0.02	Methiothepin	6.780 ± 0.078		6.788 ± 0.046
Tryptamine	2559 ± 827	1.19 ± 0.18	Methysergide	6.520 ± 0.189		6.498 ± 0.154
DOI	2970 ± 592	0.89 ± 0.06	Metergoline	5.904 ± 0.013		5.777 ± 0.033
5-Me-OT	3151 ± 1041	1.02 ± 0.06	Rauwolscine	5.674 ± 0.111		5.427 ± 0.092
DPAT	3333 ± 310	0.99 ± 0.11	• Kd value of [3H] labelled 5-HT for 5-HTR1E in human cortex tissues was 5.3 nM. (Leonhardt et al., 1989) [8] • Kd values in HEK 293 cells, either transiently or stably expressing 5-HTR1E, or from human frontal cortex for [3H]5-HT binding was 7.82 ± 0.02, 8.15 ± 0.09, and 8.17 respectively. (McAllister et al., 1992) [27] • (Kd) of [3H]5-HT was 9.7 ± 1.5 Nm for cloned human 5-HTR1E. (Zgombick et al., 1992) [28] • Kd values for [3H] labeled 5-HT were 5.691 ± 0.617 and 6.188 ± 0.700 nM ± for the guinea pig and the human 5-HTR1E receptors, respectively. (Bai et al., 2004) [20]			
Rauwoiscine	3434 ± 102	0. 85 ± 0.03				
Spiperone	5051 ± 689	0. 93 ± 0.04				
TFMPP	6293 ± 259	0.90 ± 0.07				
5-CT	7875 ± 284	0.82 ± 0.03				

3H labelled 5-HT Binding studies with cloned human and guinea pig 5-HTR1E show that 5-HTR1E receptor from both the species has similar pharmacological profile with Kd values of 5.6 and 6.1 nM respectively [20] (Table 3). In AV-12 cells, dose dependent 5-HT stimulation of [35S] GTPγS binding to the guinea pig 5-HTR1E derived an EC50 value of 13.6 nM, very similar to that of the human 5- HTR1E receptor (13.7 nM). Guinea pig 5-HTR1E also showed activation with ergonovine, α-methyl-5-HT, 1-naphthylpiperazine, methysergide, tryptamine, and 1-(2,5-dimethoxy-4-iodophenyl)-2-aminopropane (DOI) while methiothepin displayed antagonist activity [20] (Table 3). In another report on human 5-HTR1E, methiothepin was suggested to be a weak antagonist for serotonin binding site on 5-HTR1E as the effect was nonspecific since it was also able to inhibit forskolin stimulated cAMP in control BS-C-1 cells [40]. In NIH3T3 cells, 10 μM methiothepin was able to block the effect of BRL54443 (5-HTR1E agonist) on transiently expressed human 5-HTR1E [41] but in our experiments up to 30 μM methiothepin failed to antagonize the 5-HT mediated effects in human 5-HTR1E expressing HEK293 cells.

### Pharmacological comparison of 5-HTR1E with other 5-HT1 receptors

With 58% overall sequence homology with 5-HTR1E, 5-HTR1F is the closest member of 5HT1 family and these Gαi coupled receptors share pharmacological properties and second messenger systems but low affinity of 5-HTR1E for sumatriptan sets it apart from the 5-HTR1F [17, 42]. 5-HTR1F has several selective agonists like LY573144 (Lasmiditan), LY334370 and LY344864 and some of them are already in clinical trial for treatment of migraine [17, 43, 44]. On the other hand, BRL53444, a mix 5-HTR1E/F agonist is the only compound which can be used to study 5-HTR1E functions with confidence [11, 12, 19, 25, 41, 45]. Another 5-HTl family member close to 5-HTR1E receptors is 5HT1D which is ~ 66% identical in the transmembrane domains regions but still shows very different pharmacological properties. Pharmacological comparison between 5-HTR1D and 5-HTR1E receptors indicates that sequences in the 6^th^ and 7^th^ transmembrane domains (TM) are responsible for the low affinity between 5-CT and 5-HTR1E. More detailed analysis revealed that the differences in two amino acids in 6^th^ TM domain, Isoleucine 333 and Serine 334 in the 5-HT1D receptor with Lysine 310 and Glutamate 311 in the 5-HTR1E receptor were mainly accountable for the differential affinities of some ligands or inhibitors [42].

Rat 5-HTR1B [46] and human 5-HTR1A [47] receptors show high affinity for some β-adrenergic ligands like propranolol and pindolol and site-directed mutagenesis studies on these 5-HT1 receptor suggested that this pharmacological intersection between adrenergic and serotonin receptors is due to a conserved asparagine (N) residue in the seventh transmembrane domain of these receptors. Unlike these 5-HT1 receptors, 5-HTR1E has little affinity for β-adrenergic ligands due to the replacement of this asparagine (N) residue with (threonine (T330) in its 7th transmembrane domain [48].

### 5-HTR1E signaling pathways

**Fig. 1:**
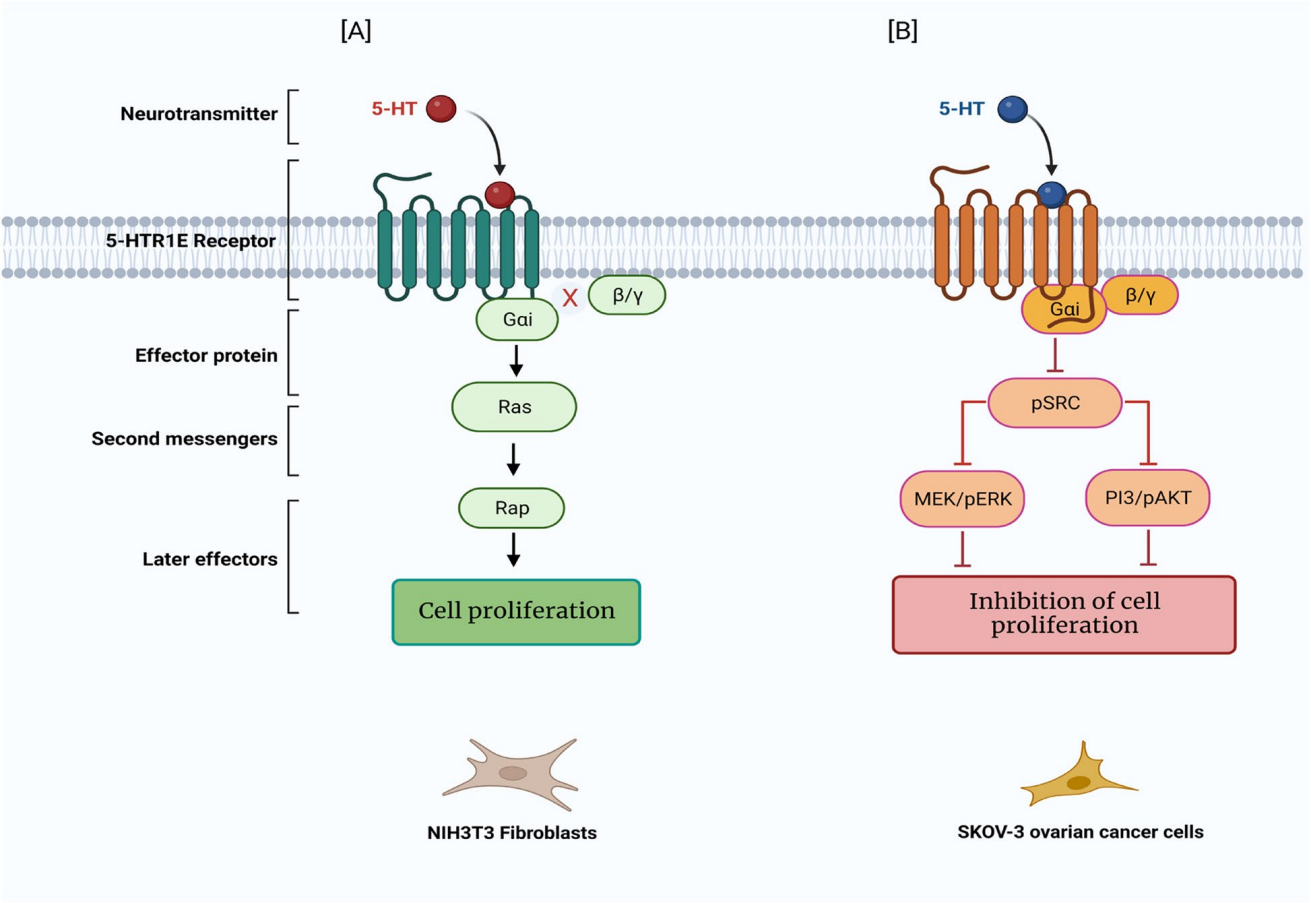
A summary of pathways affected by 5-HTR1E serotonin receptor activation. (**A**) Gαi-mediated RAS-Rap signaling pathway in serotonin (5-HT) induced 5-HTR1E overexpressing NIH3T3 mouse cells is shown on the left side. (**B**) 5-HTR1E-mediated signaling pathway which inhibit SRC/ERK/AKT signaling followed by proliferation in ovarian cancer cells is depicted on the right side. Created with BioRender.com

Like the other 5-HT1 family members, 5-HTR1E is also a Gαi coupled receptor which is negatively linked to classical cAMP pathway and works via inhibition of adenyl cyclase [20, 25–28]. At first, Ltk-cells transfected with human 5-HTR1E and tested for adenylyl cyclase activity in the absence and presence of serotonin showed 29% inhibition while in the same report, another cell line LS31/27.9 showed almost 35% forskolin-stimulated adenylyl cyclase inhibition via 5-HTR1E [26]. Further studies in guinea pig brain tissues [25] and human 5-HTR1E overexpressing HEK293 cells [11, 12] showed inhibition of cAMP levels between 30–40% which was consistent with the previous reports. In addition to cAMP pathway, 5-HTR1E can activate RAS-RAP, SRC and ERK signaling in different cell types. Ras and Rap proteins which are small GTPases can mediate the proliferation of NIH3T3 cells upon activation by 5-HRT1E receptor [41]. This RAS-RAP activation is Gαi dependent which could be blocked by pertussis toxin (PTX) but it is PKA-independent. Interestingly, Gαi dependent RAS-RAP activation was totally independent of Gβγ as the overexpression of Gβγ scavenger BarkCT [49] did not block the 5-HTR1E induced effect on Ras-Rap proteins [41] Fig. 1A.

In SKOV-3 ovarian cancer cells 5-HTR1E knockdown significantly increased SRC phosphorylation in SKOV-3 cells while in control cells serotonin inhibited pSRC in a dose-dependent manner. 5-HTR1E knock down also activated ERK phosphorylation which was reversible with SRC or the MEK inhibitor and decreased the proliferation and colony formation functions of serotonin-5-HTR1E KO SKOV-3. Cell-cycle analysis in these cells demonstrated that the inhibition of SRC or MEK can arrest 5-HTR1E KO SKOV-3 cells in G1 phase while overexpression of 5-HTR1E in OVCAR-5 cells inhibited the SRC and ERK mediated proliferation to the similar level of SRC and ERK inhibitors. In addition to ERK pathway SRC also activated PI3/AKT pathway in 5-HTR1E KO SKOV-3 cells. These studies suggested that serotonin-5-HTR1E signaling regulates cell proliferation mainly through SRC-MEK-ERK pathway in ovarian cancer cells [19] Fig. 1B. Further, Gene sets enrichment analysis (GSEA) analysis in 5-HTR1E knock out SKOV-3 cells showed that 5-HTR1E inhibition upregulates genes of epithelial mesenchymal transition (EMT), extracellular matrix organization and metabolic pathways. EMT play essential roles in the peritoneal dissemination of OC cells and 5-HTR1E silencing upregulated many genes involved in EMT-driving transcriptional factors which ultimately increased the motility of 5-HTR1E knockout SKOV-3 cells [19].

**Fig. 2:**
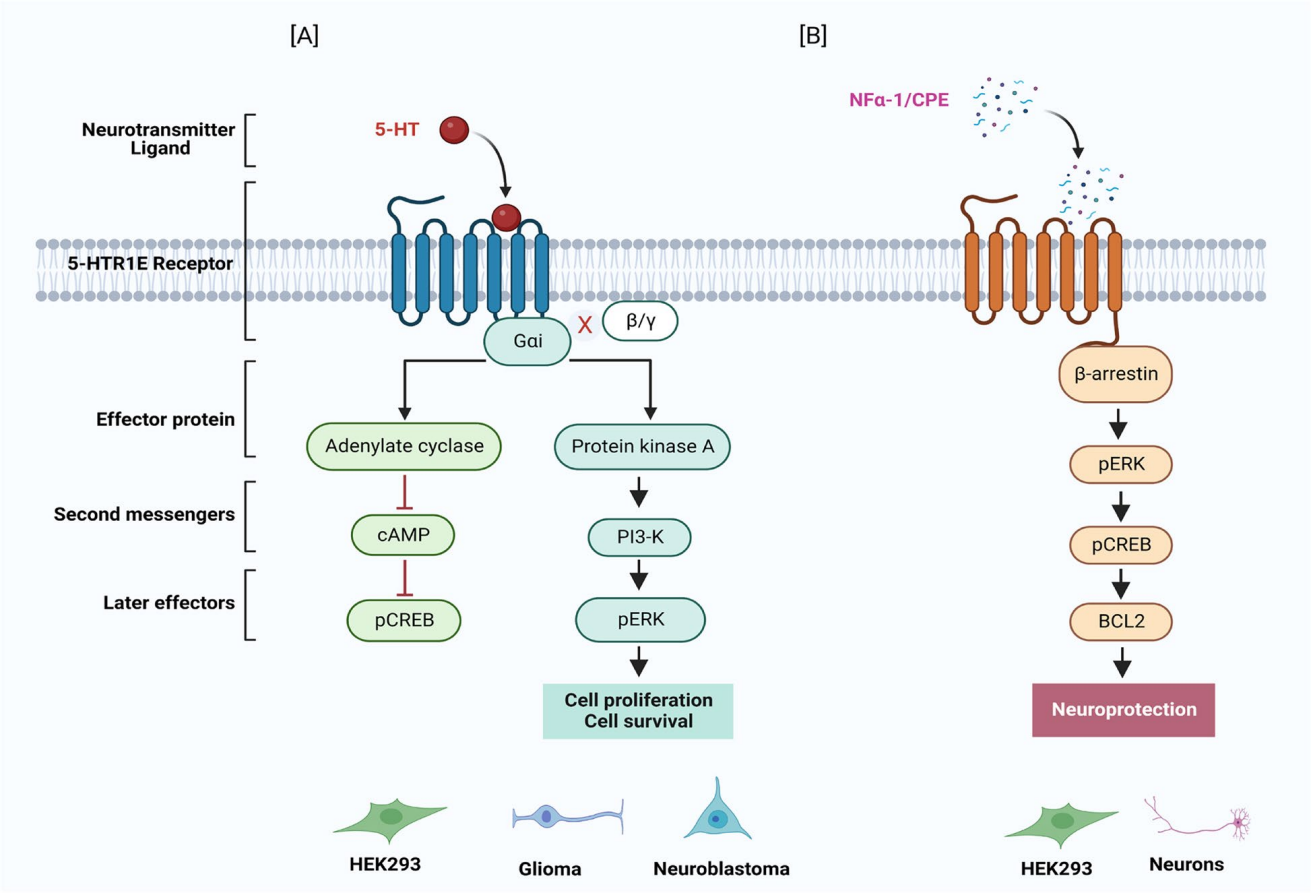
Regulation of cAMP and ERK signaling by 5-HTR1E receptor. (**A**) Upon activation with serotonin, 5-HTR1E receptor binds to Gαi protein and inhibit adenyl cyclase activity which reduces the levels of cAMP and CREB in HEK 293 cells. At the same time, it increases ERK phosphorylation via PKA/PI3-K mediated pathway and plays a role in survival or proliferation of glioma and neuroblastoma cells (**B**) Cell surface interaction between NF-α1/CPE and 5-HTR1E recruits β-arrestin to the intracellular domains of 5-HTR1E and activate pERK pathway which helps in BCL2 mediated protection of HEK293 and human neurons against oxidative stress. Created with BioRender.com

Studies in HEK293 cells have shown that human 5-HTR1E interacts with neurotrophic factor α-1/carboxypeptidase E (NFα-1/CPE) extracellularly, and this interaction recruits β-arrestin1 to the extracellular domains of 5-HTR1E which eventually activates ERK-BCL2 signaling pathway for neuroprotection and cell survival [12] Fig. 2. Serotonin was also able to activate ERK signaling in HEK293 cells via induction of human 5-HTR1E receptor. PTX sensitive, Serotonin-5-HTR1E stimulated ERK activation was Gαi-PKA-PI3K dependent while Gβγ and β-arrestin were not involved in this signaling pathway [11] which shows that 5-HTR1E can have differential coupling mechanism depending on the stimulation by a specific ligand Fig. 2. RNASeq studies in 5-HTR1E overexpressing HEK cells has shown that this serotonin receptor can also regulate the expression of various genes and pathway which are involved in important biological processes wound healing, glycoprotein metabolism, axon and mesenchymal development RNA splicing, RNA metabolism and ribosome biogenesis [11]. Based on these published reports it is evident that 5-HTR1E participates in many signaling pathways depending on its ligand/binding partner in a specific cell and tissue type where it could play a different role.

### 5-HTR1E in cell survival and neuroprotection

**Fig. 3:**
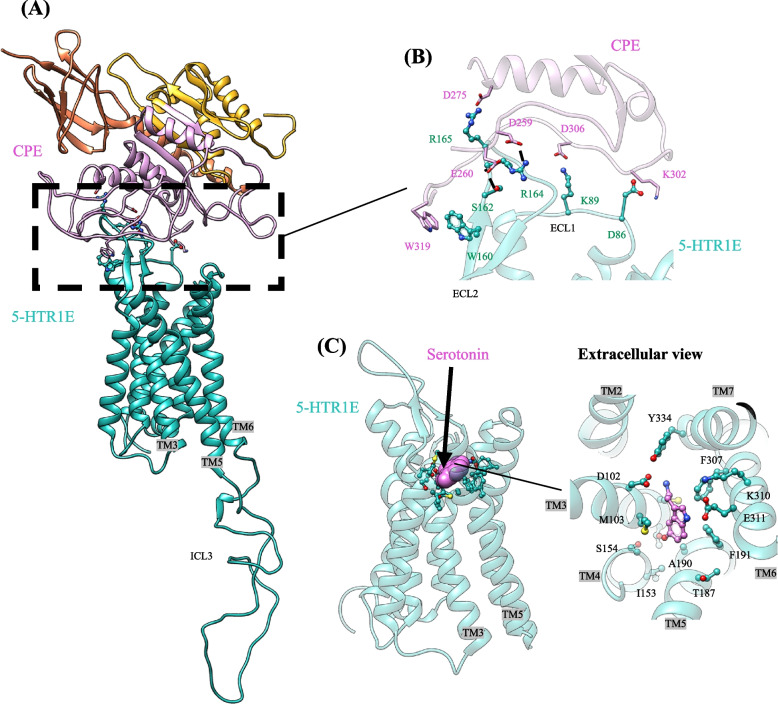
Molecular modeling of extracellular interaction between NF-α1/CPE and 5-HTR1E (**A**) The side view of binding interface between CPE and 5-HTR1E resulted from ~ 1.5 μs MD simulations colored by subunits: CPE (pink), 5-HTR1E (green). (**B**) Specific amino acids involved in tight coupling between the NF-α1/CPE and the extracellular surface of 5-HTR1E, mostly involving the polar interactions between the pair proteins. (**C**) The orthosteric serotonin binding in 5-HTR1E. Reproduced with permission from Sharma et al., 2021 [12]

Recently [12] reported that the extracellular interaction between NF-α1/CPE and 5-HTR1E promoted neuronal survival against oxidative and neurotoxic stress. In this study 5-HTR1E was able to protect HEK293 and human neuronal cells against H_2_O_2_-induced cytotoxicity or glutamate induced excitotoxicity with NF-α1/CPE pretreatment. These protective effects were mediated by cell surface interaction where NF-α1/CPE interacted with the ECL1/ECL2 (ECL: extracellular loop) of 5-HTR1E via 3 stable salt bridges comprised of K302-D86^ECL1^, D306-K89^ECL1^, D275-R165^ECL2^ (Fig. 3A, B). In contrast, the serotonin binding site is in a pocket deep in the receptor molecule (Fig. 3C). This interaction further activated a non-canonical signaling pathway where β-arrestin1 was recruited to the ICL2 and ICL3 (ICL: intracellular loop) of 5-HTR1E which further activated the ERK-CREB signaling pathway. This 5-HTR1E mediated signaling prevented the decrease in pro-survival protein BCL2 against H_2_O_2_ or glutamate induced stress and eventually led to neuroprotection and survival of these cells [12]. Colocalization of 5-HTR1E and NF-α1/CPE in the same pyramidal neurons in the CA1, CA2, and CA3 regions of the hippocampus also shows the possibility of functional interaction between NF-α1/CPE and 5-HTR1E in vivo*.* Moreover, gene knockdown studies on neuroblastoma SHSY-5Y and glioma U118 cells showed that inhibition of 5-HTR1E expression can reduce the growth of these cells by disrupting the expression of cell cycle related genes [11]. Collectively these results provide evidence that 5-HTR1E receptor is critical for the survival of nervous system cells, and plays a critical role in neuroprotection.

### 5-HTR1E in cancer

5-HTR1E gene and protein expression has been reported in various cancer cells and tissues [19, 50–53] (ref. 49, review) where it can regulate cell survival and proliferation depending on signaling pathway and coupling with second messenger in a specific cell or tissue type. In ovarian cancer (OC) patients, significantly decreased expression of 5-HTR1E was correlated with poor clinical outcome and silencing of 5-HTR1E in SKOV3 OC cells promoted cell proliferation and epithelial mesenchymal transition (EMT) via pSRC pathways. Furthermore, chronic stress induced OC growth and peritoneal dissemination was inhibited by specific 5-HTR1E agonist BRL54443 and SRC inhibitor which shows that 5-HTR1E works as a tumor suppressor in OC [19]. Based on the radioligand binding experiments in neuroblastoma samples from childrens, it was speculated that the 5-HTR1E could serve as possible markers for human neuroblastoma, as all 13 tumors examined in this report were positive for 5-HTR1E binding [52]. In neuroblastoma SHSY-5Y cells, 5-HTR1E knockdown inhibited pERK and pAKT pathways which led to the reduction in cell cycle and survival related genes such as *cMYC, cyclin D1, Cyclin E* and *BCL2*. Survival/proliferation of SHSY-5Y and glioma U118 cells was also inhibited after 5-HTR1E KO which is opposite to OC and could be attributed to differential coupling and signaling pathways in these cells [11]. These studies on 5-HTR1E in different cancer types indicate that it can regulate expression of many genes via various signaling pathways involved in tumor progression.

## Conclusions and future directions

This review summarizes the published reports on the structural, pharmacological, and molecular characteristics of 5-HTR1E. The existing data shows that 5-HTR1E receptor express in a wide variety of cell and tissue types where it can activate different signaling pathways like cAMP, ERK, AKT, SRC, RAS-RAP and regulate important biological functions. Also, in addition to the classical 5-HT1 family pathways, 5-HTR1E can also activate a non-canonical pathway, β-arrestin-ERK. It is evident that 5-HTR1E serotonin receptor plays a physiological role in neuronal stress and its expression is critical in ovarian and neuroblastoma cancers. Most of the published reports on 5-HTR1E are about cloning and identification, without any functional data. Only a few recent studies highlighted the functional role of 5-HTR1E from which one can discuss the physiological importance of this receptor. Absence of 5-HTR1E gene in small animals and unavailability of specific agonist and antagonist makes it a difficult target to study. Future studies should focus on in vivo experiments using various animal models such as guinea pig and primates to knock out 5-HTR1E and test the physiological role of the receptor in neurological function and cancer. Since 5-HTR1E can be activated with more than one ligand, the development of small molecule agonists and antagonists specific for the serotonin or CPE/NFα1 binding domains would be an important step to dissect the functions of 5-HTR1E in human neurons and cancer cells in vitro. Such studies can then be extended to in vivo animal models. This approach could potentially lead to the development of a drug targeting this receptor for treatment of diseases such as cancer and neurodegenerative disorders, through inhibiting tumor cell survival, or activating neuroprotection, respectively. Based on the available data, this review provides collective evidence that 5-HTR1E is an important GPCR, with the potential of being a prognostic and therapeutic target in various diseases.

## Data Availability

Not applicable.
